# Investigating the Effect of Increased TMEM106B in a Tauopathy Model

**DOI:** 10.1002/alz70855_107573

**Published:** 2025-12-24

**Authors:** Adulfo Anaya Amador, Isabella Aguirre‐Lamus, Dylan Lawrence Timperman, Peter Kim, Joanna L Jankowsky, Ismael Al‐Ramahi

**Affiliations:** ^1^ Baylor College of Medicine, Houston, TX, USA; ^2^ Jan and Dan Duncan Neurological Research Institute, Texas Children's Hospital, Houston, TX, USA; ^3^ Center for Alzheimer's and Neurodegenerative Diseases ‐ BCM, Houston, TX, USA

## Abstract

**Background:**

TMEM106B is a transmembrane protein localized to the lysosome and plays important roles in the transport, maturation and biogenesis of lysosomes. TMEM106B fragments have been found to accumulate in the aging brain, brains of Alzheimer's (AD) patients, and other neurodegenerative disorders. Additionally, variants in the TMEM106B gene have been linked to the risk of multiple neurodegenerative diseases, namely frontotemporal lobar degeneration with TDP‐43 aggregates (FTLD‐TDP), AD, and hypomyelinating leukodystrophy. Previous research has shown that TMEM106B knockout accelerated cognitive decline, hind limb paralysis, Tau pathology, and neurodegeneration in the brains of PS19 P301S Tau mice. Although these findings suggest TMEM106B's protective role in the context of tauopathy, assessing the effect of increasing TMEM106B levels in cells has proven difficult, as more TMEM106B leads to similar lysosomal dysfunction phenotypes as its loss.

**Method:**

To probe the effect of increasing TMEM106B levels we generated a transgenic Drosophila strain expressing human TMEM106B. We conducted age‐dependent behavioral experiments to determine the effects of TMEM106B expressed either alone or in the presence of human Tau. Immunofluorescence and biochemical studies were done to determine the localization of TMEM106B in the CNS and its effect on Tau levels and endolysosomal markers.

**Result:**

We show that TMEM106B overexpression attenuates human Tau‐induced neuronal dysfunction and is not toxic on its own, assessed as motor performance impairments as a function of age. We found an accumulation of TMEM106B protein in the CNS of Tau‐expressing animals, compared to controls. Immunofluorescence studies determined that TMEM106B exhibits a diffuse distribution in neurons, but when co‐expressed alongside Tau, TMEM106B is found in vesicle‐like structures that accumulate with age and colocalize with various endolysosomal and autophagy markers. Similar vesicular structures and colocalization were not seen in TMEM106B/+ flies not expressing human Tau, further linking Tau and TMEM106B accumulation and interaction. To expand, we performed genetic interaction experiments between TMEM106B and several endolysosomal genes, which showed that increasing TMEM106B levels mitigated the neuronal dysfunction phenotypes induced by loss of two of these genes.

**Conclusion:**

Our results support a neuroprotective role for TMEM106B in tauopathy by partially mitigating Tau‐induced lysosomal dysfunction.

